# Correlation between Candidate Single Nucleotide Variants and Several Clinicopathological Risk Factors Related to Breast Cancer in Jordanian Women: A Genotype-Phenotype Study

**DOI:** 10.7150/jca.33857

**Published:** 2019-08-08

**Authors:** Laith N. AL-Eitan, Doaa M. Rababa'h, Mansour A. Alghamdi, Rame H. Khasawneh

**Affiliations:** 1 Department of Applied Biological Sciences, Jordan University of Science and Technology, Irbid 22110, Jordan; 2 Department of Biotechnology and Genetic Engineering, Jordan University of Science and Technology, Irbid 22110, Jordan; 3 College of Medicine, King Khalid University, Abha, Saudi Arabia; 4 Department of Hematopathology, King Hussein Medical Center (KHMC), Jordan Royal Medical Services (RMS), Amman 11118, Jordan

## Abstract

This study aim to investigate the association of breast cancer risk and prognostic factors with single nucleotide variants of the *BRCA1*, *BRCA2*, *DAPK1, MMP9, TOX3*, and *TP53* genes in Jordanian women. Blood samples were collected from 230 Jordanian breast cancer patients for use in DNA extraction followed by genotyping and subsequent statistical analysis. We found that two single nucleotide variants (SNVs) of the *BRCA2* gene, namely rs1799944 and rs766173, were significantly associated with breastfeeding status. Likewise, the rs11141901 and rs1041326 SNVs of the *DAPK1* gene were linked with co-morbidity (*p-value* = 0.002) and family history of BC (*p-value* = 0.015), while the rs1045042 SNV of the same gene was associated with both allergy (*p-value* = 0.001) and family history of BC (*p-value* = 0.02). Tumor differentiation was correlated with the *DAPK1* SNVs rs11141901 (*p-value* = 0.041) and rs1041326 (*p-value* = 0.005). Additionally, the rs2250889 SNV of the *MMP9* gene was significantly associated with HER2 status, whereas the *TP53* SNVs rs12951053 and rs1042522 were associated with age at menarche (*p-value* = 0.043) and breastfeeding status (*p-value* = 0.013), respectively. In contrast, the *TP53* SNV rs2287497 was significantly linked to age at first pregnancy (*p-value* = 0.001), smoking (*p-value* = 0.041), and axillary lymph node status (*p-value* = 6e^-4^). No such association was found for the *BRCA1* and *TOX3* SNVs. The current findings suggest significant associations between certain SNVs and breast cancer risk and prognosis in Jordanian women.

## Background

In recent years, the burden of disease in Jordan has shifted away from infectious illnesses and towards non-communicable diseases like cancer, the latter of which is responsible for 14% of all Jordanian deaths 1. Cancer rates are exacerbated by the increased prevalence of risk factors for the disease as well as rising life expectancies among the Jordanian population 2. Cancer risk factors can be broadly divided into those that are preventable, which are influenced by lifestyle and environment, and those that are unpreventable, such as age, family history, and individual genetic profiles 3. Preventable risk factors that are notable among Jordanians are obesity and tobacco usage, each affecting nearly one-third of the population 4,5. Similarly, non-preventable risk factors in the form of cancer-associated genetic mutations have been identified in Jordanians 6. Despite this, genetic counselling remains under-utilized by the majority of the population, and the high rate of consanguineous unions in Jordanian society serves only to increase the risk of disease 7,8.

In 2016 the incidence of breast cancer (BC) among Arab women was (28/100,000) which is lower than the global mean (46/100,000) 9. Among Jordanian women, (BC) is the most common type of cancer, accounting for two out of five female cancers and 12.5% of all deaths 10,11. In Jordan; 37.3% 12 of all female cancers are diagnosed as BC, this percentage is similar to that in morocco (34.3%), Tunisia (31.9%), Algeria (37%) 13, Egypt (38.8%) 14, and Lebanon (38.2%) 15 but higher than those in Libya (23.2%) and Saudi Arabia (22.4%) 13.

Reported risk factors for BC in Jordanians include postmenopausal obesity, breast trauma, irregular menstruation, and usage of fertility drugs, oral contraceptives, or hair dyes, while protective factors involve sufficient levels of physical activity and fruit/vegetable intake 16-18. Mutations in BC-associated genes have been investigated in Jordanian patients, and significant correlation has been found for certain single nucleotide variants (SNVs) of the *BRCA1*, *BRCA2*, *DAPK1, MMP9,* and *TOX3* genes 19,20.

It is imperative that guidelines for BC treatment be tailored for the Jordanian population, as the age of BC diagnosis for Arab women, including Jordanians, occurs almost a decade earlier than their Western counterparts 21.

Other than risk factors, prognostic factors encompassing a range of molecular subtyping and pathologic features play a major role in BC treatment outcome 22. Molecular subtyping of BC utilizes estrogen, progesterone, and human epidermal growth factor receptor 2 (HER2) status to divide the disease into four subtypes: luminal A, luminal B, triple negative, and HER2-positive (Table 1) 23. In Jordanian patients, the molecular subtype of BC differed depending on age, with 72% of women older than 50 and 42% of women younger than 50 having the luminal A subtype 24. In another study of 752 Jordanian BC patients, triple negative BC was identified only in women under the age of 40, while estrogen receptor expression was documented in the majority of cases 25. Likewise, certain pathological features such as invasiveness, nodal involvement, tumor morphology, and tumor stage have been investigated in Jordanian BC patients, with the majority of cases being invasive carcinomas and tumor stage being the only statistically significant influence on 5-year survival rates 25,26.

Despite the relatively high prevalence of the disease, rates of BC screening have been historically low in Jordan due to the cultural stigma surrounding cancer as well as socioeconomic inequalities 27,28. In order to help alleviate this problem, it is important to understand how the preventable and non-preventable BC risk factors interplay to affect the disease's prognosis. Therefore, the primary objective of this study is to determine the extent of association between certain risk and prognostic factors for BC and previously reported SNVs in the cancer-associated *BRCA1*, *BRCA2*, *DAPK1, MMP9, TOX3*, and *TP53* genes.

## Materials and Methods

### Experimental Design and Population

Blood samples (5 ml) were collected from 230 Jordanian BC patients recruited from the Jordanian Royal Medical Services Hospital in Amman, Jordan. All patients gave written informed consent prior to their participation, and this study was ethically approved by the Institutional Review Board (IRB) at Jordan University of Science and Technology.

### Selection of candidate Single Nucleotide Polymorphisms (SNPs)

We selected a set of SNPs within candidate genes involved specifically in breast cancer from the PharmGKB database (http://www.pharmgkb.org) which provides an overview of significant polymorphisms involved in tumorgenesis processes. In addition, over than 60 genetic variants have been identified as predictable markers for breast cancer and the majority of them are involved in oncogenesis 29, 30. The selected SNPs within *BRCA2, DAPK1, MMP9*, and *TP53* genes were chosen through extensive review of a variety of sources and from reported polymorphisms associated with different types of cancers including breast cancer 19, 20, 29, 30.

### SNV Genotyping

The Wizard® Genomic DNA Purification Kit (Promega Corp., USA) was used to extract genomic DNA from the blood samples. The integrity and concentration of the extracted DNA was then confirmed by agarose gel electrophoresis and the Nano-Drop ND-1000 UV-Vis Spectrophotometer (BioDrop, UK). 20 ng/μl samples were then shipped on wet ice to the Australian Genome Research Facility's (AGRF) Melbourne node for genotyping analysis via the Sequenom MassARRAY® system (iPLEX GOLD) (Sequenom, USA). The details of SNP genotyping and the description of study cohort were summarized in early published study by AL-Eitan et al (2017) 20.

### Statistical Analysis

The χ^2^ test was performed to carry out the genotype-phenotype analyses in the present study using the Statistical Package for the Social Sciences (SPSS), version 25.0 (SPSS, Inc., Chicago, IL). The odds ratio (OR) was also calculated using binary logistic regression with 95% confidence intervals (CI). P-values were considered to be significant if they were less than 0.05.

## Results

### Demographics of Experimental Population

74.6% of the 230 Jordanian BC patients were married with the average age (± SD) being 53.19 ± 12.777 years. 67.3% of the participants had breastfed at some point in their lives. In cross-sectional study, Khassawneh et al (2006) demonstrated that the initiation rate of breastfeeding of 88.6% which is higher than it reported in the USA for the same ages. However, it was less than breast feeding rates in other Middle Eastern countries 31. While, the rate of exclusive breastfeeding (2013- 2015) at 6 weeks among Jordanian mothers was 25.5% among, and this rate dropped to 2.1% at 6 months 32. This decline in breastfeeding practicing among Jordanian women can be attributed to increased mothers' ages, employed mothers, and lack of breastfeeding education during pregnancy and after birth.

Likewise, the average ages of BC diagnosis and menopause were 50.90 ± 12.2 and 48.50 ± 5.3 years, respectively. Regarding co-morbidities, 36.3% of BC patients suffered from other complications such as hypertension, coronary artery disease, asthma, and diabetes mellitus. In terms of pathological BC features, 74.7% of all cases had been diagnosed with invasive ductal carcinoma compared to the 7.4% that were found to have in-situ ductal carcinoma. With regard to hormone receptor status, estrogen and progesterone receptors were found on the malignant cells of 77.5% and 68.8% of patients, respectively, while 35.4% of patients were positive for HER2 expression.

### Association of BRCA1 and BRCA2 SNVs with Prognostic and Risk Factors for BC

None of the investigated *BRCA1* SNVs showed any significant association with the risk factors for and pathological features of BC in Jordanian patients (Tables 3a and 3b). In contrast, the *BRCA2* SNVs rs1799944 and rs766173 were both significantly associated with breastfeeding status, having p-values of 0.041 and 0.002, respectively. However, like *BRCA1*, no *BRCA2* SNV was found to be associated with pathological BC features in Jordanian patients (Tables 2a and 2b).

### Association of DAPK1 and MMP9 SNVs with Prognostic and Risk Factors for BC

Tables 3a and 3b illustrate the relationship between certain *DAPK1* and *MMP9* SNVs and clinical-pathologic BC features. The *DAPK1* SNV rs11141901 showed a strong association with co-morbidity (*p-value* = 0.002), while the rs1045042 SNV of the same gene was correlated with both allergy (*p-value* = 0.001) and family history of BC (*p-value* = 0.02). Family history of BC was also significantly associated with the *DAPK1* SNV rs1041326 (*p-value* = 0.015). Concerning pathological features, tumor differentiation was significantly associated with the *DAPK1* SNVs rs11141901 (*p-value* = 0.041) and rs1041326 (*p-value* = 0.005). For the investigated *MMP9* SNVs, only rs2250889 showed an association with any clinical or pathological factor, namely HER2 status (*p-value* = 0.044).

### Association of TOX3 and TP53 SNVs with Prognostic and Risk Factors for BC

The association between the investigated *TOX3* and *TP53* SNVs and certain BC features are shown in Tables 4a and 4b. The *TOX3* SNV did not exhibit a significant relationship with any of the selected clinical and pathological BC features. In contrast, the *TP53* SNVs rs12951053 and rs1042522 were found to be significantly associated with age at menarche (*p-value* = 0.043) and breastfeeding status (*p-value* = 0.013), respectively. Additionally, the *TP53* SNV rs2287497 was linked to both age at first pregnancy (*p-value* = 0.001) and smoking (*p-value* = 0.041). The only pathological feature to be linked to any *TP53* SNV was axillary lymph node status, which was significantly associated with rs2287497 (*p-value* = 6e^-4^).

### Association of BRCA1, BRCA2, TP53, DAPK1, MMP9, and TOX3 SNVs with molecular subtype of BC

Despite the importance of molecular subtyping to BC prognosis and treatment, no association was found between these heterogenic markers and any candidate SNV in this study (Table 5).

## Discussion

Breast cancer (BC) affects two out of five Jordanian women and is responsible for one out of ten deaths in Jordan 10,11. Despite being a leading cause of morbidity and mortality, BC screening is not widespread among Jordanian women, and studies of the disease as it occurs in Jordanian women are not exhaustive 2,27,28. The fact that BC risk and prognosis is modulated by ethnic differences demonstrates that research involving non-Jordanian women cannot simply be extrapolated to Jordanians, highlighting the need for studies involving Jordanian patients 33. In the present study, the association of BC risk and prognosis with certain SNVs of the *BRCA1, BRCA2, TP53, DAPK1, MMP9 and TOX3* genes was investigated.

Mutations within high-penetrance genes; breast cancer 1 (BRCA1) and breast cancer 2 (BRCA2) impact the underlying functions of those genes as tumor suppressor genes 34. Moreover, *BRCA1* and *BRCA2* are responsible for DNA repair by homologous recombination. However, *BRCA*1/2 variants distributions are vary among populations, statistics about hereditary breast cancer among the Arabs origin are very scarce. For example, a Lebanese study was reported that 5.6% of high risk BC patients carried a deleterious *BRCA*1/2 mutations 35. Another study was conducted in Moroccan women diagnosed with BC claimed that 31.6% of familial BC was found to be associated with BRCA1 mutations^36^ while in Egypt 60% of familial BC cases were attributed to *BRCA1* mutations and approximately 26% were because of *BRCA2* mutations 37. A recent study in Jordan included 100 women diagnosed with BC reported that 20% patients had deleterious *BRCA1* or *BRCA2* mutations 19.

The tumor protein 53 (*TP53*) gene is another high-penetrance breast cancer susceptibility gene. Mutation locus (upstream and downstream) in *TP53* can influence gene function. rs1042522 SNP of TP53 has been suggested that is considered as prognostic marker associated with a low tumor grade in breast cancer 38. Another gene suggested to be involved in breast cancer is death-associated protein kinase 1 (*DAPK1*) which is known in its role of inducing cell death and recognized as a tumor suppressor gene. Mutations within *DAPK1* gene have implicated in down regulation of *DAPK1* transcription 39.

Matrix Metallopeptidase 9 (*MMP9*) is a member of the Matrix Metalloproteinases (MMPs) family and considers as metastasis-associated gene. This gene is also involved in tumor growth, invasion, carcinogenesis and angiogenesis and has been found that the activity and the expression levels increase in malignant breast tumors. These make the *MMP9* as a useful marker for BC prognosis 40. While, The TOX high-mobility group box family member 3 (*TOX3*) is classified as low-penetrance gene that plays a crucial function in chromatin structure alteration. It has been also suggested that an amplified expression of TOX3 can lead to bone metastasis 41.

Mutations in the *BRCA1* and *BRCA2* genes are perhaps the most well-known causes of hereditary BC due to their disruption of the genes' tumor-suppressing functions 34. Among Jordanian patients with a history of BC, most screened mutations were found within exon 11 of the *BRCA1* gene 42. In another study, 20% of Jordanian BC cases possessed deleterious mutations in the *BRCA1* and *BRCA2* genes, and the highest mutation rate was found in triple-negative tumors 19. Our findings show that the *BRCA1* SNVs rs8176318, rs8176265 rs3737559, rs16940, and rs799905 did not have any significant association with BC risk and prognosis in Jordanians. On the other hand, breastfeeding status was significantly associated with both the *BRCA2* SNVs rs1799944 and rs766173.

Like the *BRCA1* and* BRCA2* genes*,* the death-associated protein kinase 1 (*DAPK1*) gene possesses tumor-suppressive properties, and mutations in this gene are particular associated with triple-negative BC 43. In a previous study, the *DAPK1* SNV rs11141901 was found to be significantly linked to increased BC risk in Jordanians 20. The findings of the current study show that the *DAPK1* SNV rs1045042 is significantly associated with allergy and family history of BC, while the *DAPK1* SNV rs1041326 is correlated with family history of BC and tumor differentiation. In addition, the *DAPK1* SNV rs11141901 was significantly associated with both co-morbidity and tumor differentiation. On a similar note, the matrix metallopeptidase 9 (*MMP9*) gene has also been implicated in triple-negative BC risk and development due to its angiogenic and matrix remodeling functions 44. It has been reported that the *MMP9* SNV rs6065912 was significantly associated with increased BC risk in Jordanians 20. In contrast, our findings show that the *MMP9* SNV rs2250889 was only significantly associated with HER2 status.

Likewise, the tumor protein p53 (*TP53*) gene has long been known its multitude of anticancer functions and mechanisms, and mutations in this gene have been reported to increase the metabolic capacity of BC cells 45. Our findings show that the *TP53* SNVs rs12951053 and rs1042522 were significantly associated with age at menarche and breastfeeding status, respectively, while the *TP53* SNV rs2287497 was linked to age at first pregnancy, smoking, and axillary lymph node status. In contrast to the *TP53* gene, the TOX high mobility group box family member 3 (*TOX3*) gene, which is involved in calcium-dependent transcription, has only recently been implicated in BC development 46. In Jordanians, the *TOX3* SNV rs1420546 was found to be associated with increased BC risk by a prior study 26. Contrastingly, the present study did not demonstrate significant association between the *TOX3* SNV rs1420546 and any factors related to BC risk and prognosis.

Molecular subtyping of BC differentiates between high-risk and low-risk patients, improving overall prognosis and therapeutic outcome 47. In a study of 193 Jordanian patients, the most common BC subtype was found to be luminal A 34. In contrast, the present findings do not show any significant association between the investigated *BRCA1, BRCA2, DAPK1, MMP9, TOX3*, and *TP53* SNVs and molecular subtype of BC. However, more research needs to be carried out regarding BC subtypes in Jordanians due to the fact that each subtype is associated with specific age-distribution patterns 48.

Conclusively, the current findings illustrate the relationship between specific *BRCA2, DAPK1, MMP9*, and *TP53* SNVs and BC risk and prognosis in Jordanian women. Our findings shed some light on the nature of BC as it occurs among Jordanian women and could be used in awareness and prevention initiatives. Moreover, identifying prognostic factors that can predict the risk of cancer development and progression is an urgent need in clinical practice. Determining variants involved in breast cancer patients' prognosis can also help in stratifying patients in clinical trials and lead to identifying the most effective therapy to provide patients with personalized medicine treatment. Limitations of the present study include the relatively small sample size and the lack of data related to healthy controls. Future lines of research should incorporate a larger sample cohort comprising both cases and controls to understand the association of different genes with BC risk and prognosis.

## Figures and Tables

**Table 1:** Breast cancer (BC) classification according to molecular subtype

Molecular subtype	Estrogen receptor (ER)	Progesterone receptor (PR)	HER2
Luminal A	ER(+) and /or PR(+)		(-)
Luminal B	ER(+) and /or PR(+)		(+)
Triple-negative	(-)	(-)	(-)
HER+	(-)	(-)	(+)

**Table 2a:** Association between different *BRCA1* and *BRCA2* SNVs and risk factors for breast cancer (BC)

Risk factor	*BRCA1*					*BRCA2*	
	rs8176318	rs8176265	rs3737559	rs16940	rs799905	rs1799944	rs766173
Age at BC diagnosis **	0.850^a^ 0.035^b^	0.899^a^ 0.016^b^	0.532^a^ 0.390^b^	0.781^a^ 0.08^b^	0.196^a^ 1.672^b^	0. 733^a^ 0.116^b^	0.242^a^ 1.369^b^
Age at first pregnancy **	0.454^a^ 0.560^b^	0.401^a^ 0.705^b^	0.710^a^ 0.138^b^	0.406^a^ 0.690^b^	0.185^a^ 1.757^b^	0.462^a^ 0.541^b^	0.346^a^ 0.888^b^
Age at menarche **	0.971^a^ 0.001^b^	0.931^a^ 0.007^b^	0.589^a^ 0.291^b^	0.651a 0.204^b^	0.762^a^ 0.091^b^	0.152^a^ 2.052^b^	0.141^a^ 2.167^b^
Age at menopause **	0.467^a^ 0.529^b^	0.303^a^ 1.06^b^	0.330^a^ 0.948^b^	0.302^a^ 1.06^b^	0.301^a^ 1.07^b^	0.763^a^ 0.09^b^	0.457^a^ 0.553^b^
Allergy *	0.985^a^ 0.0003^b^	0.881^a^ 0.022^b^	0.401^a^ 0.705^b^	0.989^a^ 0.0002^b^	0.887^a^ 0.020^b^	0.347^a^ 0.884^b^	0.525^a^ 0.404^b^
Breastfeeding status *	0.760^a^ 0.093^b^	0.716^a^ 0.132^b^	0.283^a^ 1.153^b^	0.608^a^ 0.263^b^	0.921^a^ 0.01^b^	0.041^a^ 4.176^b^	0.002^a^ 9.55^b^
Body mass index **	0.458^a^ 0.550^b^	0.391^a^ 0.735^b^	0.489^a^ 0.478^b^	0.223^a^ 1.485^b^	0.989^a^ 0.0002^b^	0.453^a^ 0.563^b^	0.325^a^ 0.968^b^
Co-morbidity *	0.489^a^ 0.478^b^	0.780^a^ 0.078^b^	0.701^a^ 0.147^b^	0.706^a^ 0.142^b^	0.202^a^ 1.628^b^	0.214^a^ 1.544^b^	0.147^a^ 2.10^b^
Family history *	0.499^a^ 0.457^b^	0.423^a^ 0.642^b^	0.592^a^ 0.3^b^	0.388^a^ 0.745^b^	0.604^a^ 0.269^b^	486^a^ 0.485^b^	0.531^a^ 0.392^b^
Smoking *	0.689^a^ 0.16^b^	0.671^a^ 0.180^b^	0.287^a^ 1.13^b^	0.703^a^ 0.145^b^	0.914^a^ 0.012^b^	0.125^a^ 2.354^b^	0.165^a^ 1.928^b^

a: P-Value, b: Chi squared value.* Pearson's chi-squared test was used to determine genotype-phenotype association.** Analysis of variance (ANOVA) was used to determine genotype-phenotype association. P-Value <0.05 is considered as significant.

**Table 2b:** Association between different *BRCA1* and *BRCA2* SNVs and prognostic factors for breast cancer (BC)

Prognostic factor	*BRCA1*					*BRCA2*	
	rs8176318	rs8176265	rs3737559	rs16940	rs799905	rs1799944	rs766173
Axillary lymph nodes *	0.411^a^ 0.675^b^	0.680^a^ 1.883^b^	0.362^a^ 0.830^b^	0.620^a^ 0.245^b^	0.221^a^ 1.498^b^	0.763^a^ 0.090^b^	0.659^a^ 0.194^b^
Estrogen receptor status *	0.720^a^ 0.128^b^	0.687^a^ 0.162^b^	0.491^a^ 0.474^b^	0.675^a^ 0.175^b^	0.798^a^ 0.065^b^	0.902^a^ 0.015^b^	0.868^a^ 0.027^b^
HER2 *	0.323^a^ 0.976^b^	0.640^a^ 0.218^b^	0.921^a^ 0.010^b^	0.559^a^ 0.341^b^	0.449^a^ 0.596^b^	0.556^a^ 0.346^b^	0.687^a^ 0.162^b^
In-situ vs. Invasive *	0.3801^a^ 0.770^b^	0.683^a^ 0.166^b^	0.576^a^ 0.312^b^	0.282^a^ 1.157^b^	0.721^a^ 0.127^b^	0.352^a^ 0.866^b^	0.341^a^ 0.906^b^
Lymph node involvement *	0.605^a^ 0.267^b^	0.371^a^ 0.800^b^	0.543^a^ 0.37^b^	0.276^a^ 1.187^b^	0.432^a^ 0.617^b^	0.582^a^ 0.303^b^	0.124^a^ 2.366^b^
Progesterone receptor status *	0.765^a^ 0.089^b^	0.688^a^ 0.161^b^	0.621^a^ 0.244^b^	0.735^a^ 0.114^b^	0.786^a^ 0.073^b^	0.403^a^ 0.699^b^	0.901^a^ 0.015^b^
Tumor differentiation *	0.831^a^ 0.045^b^	0.897^a^ 0.017^b^	0.407^a^ 0.687^b^	0.942^a^ 0.005^b^	0.385^a^ 0.755^b^	0.879^a^ 0.023^b^	0.873^a^ 0.025^b^
Tumor size **	0.406^a^ 1.803^b^	0.516^a^ 1.323^b^	0.897^a^ 0.217^b^	0.521^a^ 0.411^b^	0.453^a^ 1.584^b^	0.921^a^ 0.01^b^	0.436^a^ 1.66^b^
Tumor stage *	0.510^a^ 0.434^b^	0.138^a^ 2.2^b^	0.084^a^ 2.986^b^	0.156^a^ 2.013^b^	0.211^a^ 1.565^b^	0.486^a^ 0.485^b^	0.706^a^ 0.142^b^

a: P-Value, b: Chi squared value. * Pearson's chi-squared test was used to determine genotype-phenotype association. ** Analysis of variance (ANOVA) was used to determine genotype-phenotype association. P-Value <0.05 is considered as significant.

**Table 3a:** Association between different *DAPK1* and *MMP9* SNVs and risk factors for breast cancer (BC)

Risk factor	*DAPK1*		*MMP9*	
	rs11141901	rs1041326	rs1045042	rs2250889
Age at BC diagnosis **	0.987^a^ 0.0003^b^	0.263^a^ 1.253^b^	0.503^a^ 0.448^b^	0.488^a^ 0.480^b^
Age at first pregnancy **	0.474^a^ 0.512^b^	0.761^a^ 0.092^b^	0.703^a^ 0.145^b^	0.683^a^ 0.166^b^
Age at menarche **	0.392^a^ 0.7327^b^	0.935^a^ 0.006^b^	0.315^a^ 1.01^b^	0.681^a^ 0.169^b^
Age at menopause **	0.130^a^ 2.29^b^	0.593^a^ 0.285^b^	0.258^a^ 1.279^b^	0.359^a^ 0.841^b^
Allergy *	0.616^a^ 0.251^b^	0.218^a^ 1.517^b^	0.001^a^ 10.83^b^	0.203^a^ 1.621^b^
Breastfeeding status *	0.488^a^ 0.480^b^	0.738^a^ 0.111^b^	0.139^a^ 2.189^b^	0.871^a^ 0.026^b^
Body mass index **	0.856^a^ 0.032^b^	0.246^a^ 1.346^b^	0.715^a^ 0.133^b^	0.639^a^ 0.220^b^
Co-morbidity *	0.002^a^ 9.55^b^	0.387^a^ 0.748^b^	0.072^a^ 3.237^b^	0.508^a^ 0.438^b^
Family history *	0.379^a^ 0.77^b^	0.015^a^ 5.91^b^	0.020^a^ 5.412^b^	0.183^a^ 1.77^b^
Smoking *	0.373^a^ 0.793^b^	0.684^a^ 0.165^b^	0.884^a^ 0.021^b^	0.214^a^ 1.544^b^

a: P-Value, b: Chi squared value. * Pearson's chi-squared test was used to determine genotype-phenotype association. ** Analysis of variance (ANOVA) was used to determine genotype-phenotype association. P-Value <0.05 is considered as significant.

**Table 3b:** Association between different DAPK1 and MMP9 SNVs and prognostic factors for breast cancer (BC)

Prognostic factor	*DAPK1*			*MMP9*	
	rs11141901	rs1041326	rs1045042	rs2250889	rs6065912
Axillary lymph nodes *	0.863^a^ 0.029^b^	0.401^a^ 0.705^b^	0.586^a^ 0.296^b^	0.645^a^ 0.212^b^	0.565^a^ 0.331^b^
Estrogen receptor status *	0.159^a^ 1.984^b^	0.598^a^ 0.278^b^	0.152^a^ 2.052^b^	0.369^a^ 0.807^b^	0.631^a^ 0.230^b^
HER2 *	0.120^a^ 2.417^b^	0.721^a^ 0.127^b^	0.786^a^ 0.073^b^	0.044^a^ 4.05^b^	0.446^a^ 0.580^b^
In-situ vs. Invasive *	0.357^a^ 0.848^b^	0.708^a^ 0.140^b^	0.704^a^ 0.144^b^	0.540^a^ 0.375^b^	0.675^a^ 0.175^b^
Lymph node involvement *	0.718^a^ 0.130^b^	0.811^a^ 0.057^b^	0.756^a^ 0.096^b^	0.316^a^ 1.1^b^	0.406^a^ 0.69^b^
Progesterone receptor status *	0.137^a^ 2.211^b^	0.863^a^ 0.029^b^	0.571^a^ 0.321^b^	0.086^a^ 2.94^b^	0.663^a^ 0.189^b^
Tumor differentiation *	0.041^a^ 4.176^b^	0.005^a^ 7.87^b^	0.631^a^ 0.230^b^	0.822^a^ 0.05^b^	0.401^a^ 0.71^b^
Tumor size **	0.982^a^ 0.036^b^	0.368 1.99^b^	0.318^a^ 2.29^b^	0.469^a^ 1.51^b^	0.209^a^ 3.131^b^
Tumor stage *	0.442^a^ 0.591^b^	0.608^a^ 0.275^b^	0.122^a^ 2.391^b^	0.099^a^ 2.72^b^	0.805^a^ 0.060^b^

a: P-Value, b: Chi squared value. * Pearson's chi-squared test was used to determine genotype-phenotype association. ** Analysis of variance (ANOVA) was used to determine genotype-phenotype association. P-Value <0.05 is considered as significant.

**Table 4a:** Association between different *TOX3* and *TP53* SNVs and risk factors for breast cancer (BC)

Risk factor	*TOX3*	*TP53*		
	rs1420546	rs12951053	rs1042522	rs2287497
Age at BC diagnosis **	0.730^a^ 0.119^b^	0.116^a^ 2.471^b^	0.546^a^ 0.364^b^	0.814^a^ 0.055^b^
Age at first pregnancy **	0.294^a^ 1.101^b^	0.751^a^ 0.101^b^	0.267^a^ 1.232^b^	0.001^a^ 10.83^b^
Age at menarche **	0.520^a^ 0.413b	0.043^a^ 4.095b	0.423^a^ 0.642^b^	0.253^a^ 1.307^b^
Age at menopause **	0.372^a^ 0.797^b^	0.069^a^ 3.307^b^	0.637^a^ 0.222^b^	0.189^a^ 1.72^b^
Allergy *	0.546^a^ 0.364^b^	0.642^a^ 0.216^b^	0.323^a^ 0.976^b^	0.564^a^ 0.332^b^
Breastfeeding status *	0.545^a^ 0.366^b^	0.552^a^ 0.353^b^	0.013^a^ 6.169^b^	0.182^a^ 1.781^b^
Body mass index **	0.155^a^ 2.02^b^	0.274^a^ 1.197^b^	0.587^a^ 0.295^b^	0.568^a^ 0.326^b^
Co-morbidity *	0.375^a^ 0.787^b^	0.098^a^ 2.73^b^	0.953^a^ 0.003^b^	0.625^a^ 0.238^b^
Family history *	0.663^a^ 0.189^b^	0.788^a^ 0.072^b^	0.286^a^ 1.138^b^	0.544^a^ 0.368^b^
Smoking *	0.366^a^ 0.817^b^	0.207^a^ 1.59^b^	0.078^a^ 3.11^b^	0.041^a^ 4.17^b^

a: P-Value, b: Chi squared value. * Pearson's chi-squared test was used to determine genotype-phenotype association. ** Analysis of variance (ANOVA) was used to determine genotype-phenotype association. P-Value <0.05 is considered as significant.

**Table 4b:** Association between different *TOX3* and *TP53* SNVs and prognostic factors for breast cancer (BC)

Pathological BC features	*TOX3*	*TP53*		
	rs1420546	rs12951053	rs1042522	rs2287497
Axillary lymph nodes *	0.967^a^ 0.002^b^	0.251^a^ 1.318^b^	0.158^a^ 1.993^b^	6e-4^a^ 20.49^b^
Estrogen receptor status *	0.231^a^ 1.435^b^	0.956^a^ 0.003^b^	0.345^a^ 0.891^b^	0.829^a^ 0.046^b^
HER2 *	0.152^a^ 2.05^b^	0.366a 0.817^b^	0.279^a^ 1.172^b^	0.337^a^ 0.921^b^
In-situ vs. Invasive *	0.397^a^ 0.717^b^	0.308^a^ 1.04^b^	0.736^a^ 0.113^b^	0.589^a^ 0.291^b^
Lymph node involvement *	0.589^a^ 0.291^b^	0.629^a^ 0.233^b^	0.419^a^ 0.653^b^	0.631^a^ 0.230^b^
Progesterone receptor status *	0.068^a^ 3.33^b^	0.469^a^ 0.524^b^	0.228^a^ 1.453^b^	0.946^a^ 0.004^b^
Tumor differentiation *	0.791^a^ 0.070^b^	0.391^a^ 0.735^b^	0.772^a^ 0.083^b^	0.451^a^ 0.56^b^
Tumor size **	0.489^a^ 1.431^b^	0.591^a^ 1.052^b^	0.296^a^ 2.435^b^	0.646^a^ 0.873^b^
Tumor stage *	0.892^a^ 0.018^b^	0.261^a^ 1.263b	0.381^a^ 0.767^b^	0.297^a^ 1.09^b^

a: P-Value, b: Chi squared value. * Pearson's chi-squared test was used to determine genotype-phenotype association. ** Analysis of variance (ANOVA) was used to determine genotype-phenotype association. P-Value <0.05 is considered as significant.

**Table 5:** Association between different intrinsic BC subtypes and each *BRCA1*, *BRCA2*, *TP53*, *DAPK1*, *MMP9*, and *TOX3* SNV

Gene	SNV ID	Luminal A vs Luminal B vs Triple-negative
***BRCA1***	rs16940	0.442^a^ 1.633^b^
	rs799905	0.823^a^ 0.389^b^
	rs8176318	0.720^a^ 0.657^b^
	rs3737559	0.701^a^ 0.710^b^
	rs8176265	0.766^a^ 0.533^b^
***BRCA2***	rs1799944	0.456^a^ 1.571^b^
	rs766173	0.649^a^ 0.864^b^
***DAPK1***	rs11141901	0.104^a^ 4.527^b^
	rs1045042	0.804^a^ 0.436^b^
	rs1041326	0.323^a^ 2.26^b^
***MMP9***	rs6065912	0.273^a^ 2.597^b^
	rs2250889	0.183^a^ 3.397^b^
***TOX3***	rs1420546	0.065^a^ 5.467^b^
***TP53***	rs1042522	0.093^a^ 4.75^b^
	rs12951053	0.527^a^ 1.281^b^
	rs2287497	0.212^a^ 3.102^b^

a: P-Value, b: Chi squared value. P-Value <0.05 is considered as significant.

## Abbreviations

AGRF: Australian Genome Research Facility
BC: Breast Cancer
χ^2^: Chi squared value
DNA: Deoxyribonucleic acid
ER: Etrogen Receptor
GWAS: genome-wide association study
GJRMS: Jordanian Royal Medical Services
HWE: Hardy-Weinberg equilibrium
Het: Heterozygote
Hz: Homozygote
HER2: Human epidermal growth factor receptor 2 marker
IRB: Institutional Review Board
PR: Progesterone Receptor
SNVs: Single nucleotide variants
SPSS: Statistical Package for the Social Sciences
